# Co-Enzyme Q10 Supplementation Rescues Cumulus Cells Dysfunction in a Maternal Aging Model

**DOI:** 10.3390/antiox8030058

**Published:** 2019-03-08

**Authors:** Assaf Ben-Meir, Kyunga Kim, Rosanne McQuaid, Navid Esfandiari, Yaakov Bentov, Robert F. Casper, Andrea Jurisicova

**Affiliations:** 1 Lunenfeld Tanenbaum Research Institute, Mount Sinai Hospital, 25 Orde Street, Toronto, ON M5T 3H7, Canada; assaf.benmeir@gmail.com (A.B.-M.); sallykyunga.kim@mail.utoronto.ca (K.K.); rosanne.mcquaid@mail.utoronto.ca (R.M.); casper@lunenfeld.ca (R.F.C.); 2 TRIO Fertility Partners, 655 Bay St, Suite 1101, Toronto, ON M5G2K4, Canada; Navid.Esfandiari@hitchcock.org (N.E.); ybentov@junofertility.com (Y.B.); 3 Department of Physiology, University of Toronto, 1 King’s College Circle, Toronto, ON M5S 1A8, Canada; 4 Department of Obstetrics and Gynecology, University of Toronto, 92 College Street, Toronto, ON M5G 1L4, Canada

**Keywords:** aging, cumulus cells, mitochondria, apoptosis

## Abstract

Over the past four decades, due to cultural and social changes, women in the developed world have significantly delayed childbirth. This trend is even worse for patients who attend infertility clinics. It is well-known that live birth rates in women older than 35 are significantly lower than in those younger, both naturally and with assisted reproduction. Fertility decline is, in part, due to an increase in oocyte aneuploidy that leads to a reduced embryo quality, as well as an increased incidence of miscarriages and birth defects. Here we show that aging-associated malfunction is not restricted to the oocyte, as cumulus granulosa cells also display a series of defects linked to mitochondrial activity. In, both, human and mouse model, a decline in cumulus cell function due to increased maternal age is accompanied by a decreased expression of enzymes responsible for Coenzyme Q (CoQ) production, particularly Pdss2 and CoQ6. In an aged mouse model supplementation with Coenzyme Q10—a potent stimulator of mitochondrial function—restored cumulus cell number, stimulated glucose uptake, and increased progesterone production. CoQ10 supplementation might, thus, improve oocyte and cumulus cells quantity and quality, by improving the mitochondrial metabolism in females of advanced maternal age.

## 1. Introduction

Due to various cultural and social shifts occurring in the past 50 years, women in most western countries significantly postpone the decision to have a child [1]. This is reflected in a 36% increase in the proportion of first births in women in their mid- to late-30s, with an even more dramatic increase for women over 40 [2]. In numerous studies it has been well-documented that female fertility, whether natural or assisted, dramatically declines with age. While several underlying causes for this decline have been proposed, an increase in oocyte aneuploidy causing a reduced embryo developmental competence and an accompanying increase of miscarriages, is considered to be the single most significant factor.

There are several theories on the pathogenesis of ovarian senescence, including a lower energy content in the oocyte, shortening of telomeres, decrease in DNA repair capacity, alteration of epigenetic chromatin hallmarks, as well as a precocious loss of chromosome cohesion [3]. It is likely that all of these pathways decline concomitantly. We have previously proposed that decline in mitochondrial function could be one of the driving factors behind the accelerated aging of oocyte [4]. Coenzyme Q (CoQ) is an essential component of the mitochondrial electron transport chain and ATP production. While in rodents, the major isoform of CoQ is CoQ9 (number referring to a length of isoprenoid side chain), with minor and tissue-context variable levels for CoQ10, human tissues mostly contain CoQ10 (reviewed in [5]). We recently reported that the expression of enzymes involved in CoQ synthesis is reduced in immature oocytes of aged mice and older women. Treatment with CoQ10 improved oocyte mitochondrial performance, as well as ovulation and pregnancy rates in aged mice [6]. However, the oocyte is not the only cell type in the ovary affected by aging. Human cumulus cells obtained from patients of advanced maternal age display a decreased expression of several proteins involved in oxidative phosphorylation (OXPHOS), particularly NDUFA1, UQCRC1, and ATP5I [7]. In addition, granulosa cells of older patients exhibit a 50% reduction in CoQ-dependent mitochondrial respiratory complex activity [8]. Furthermore, in humans, CoQ10 levels in the follicular fluid correlate with oocyte quality, as evidenced by the resulting embryo development potential [9,10,11].

Oocyte quality depends on the cumulus cells (CC) function [12]. Oocyte growth within the cumulus–oocyte complex (COC) is contingent upon a coordinated crosstalk between the oocyte and cumulus corona radiate, and is facilitated by an exchange of molecular information, via gap junctions formed by connexin 37 [13]. The cumulus cells play a crucial nurturing role in oocyte development, providing not only growth factors and lipids, but also metabolites (pyruvate, amino acids, and nucleotides), which support the demands of growing oocytes [14,15]. Several previous studies have showed a correlation between gene expression and the function of CCs and oocyte quality [16,17,18,19].

In this study, we examined whether aging is associated with a decline in the expression of genes involved in the CoQ production and the physiological function of cumulus cells. We explored if the changes we have previously observed in the aged oocytes could also apply to cumulus cells. Our results point to the overlapping age-associated decrease in the expression of both Pdss2 and CoQ6, in both cell types.

## 2. Material and Methods

### 2.1. Animals and CoQ10 Treatment

The mice model and CoQ10 treatment has been previously described [6]. Briefly, Institute of Cancer Research ICR female mice were obtained from the Harlan Laboratories Incorporated (Mississauga, ON, Canada) or Taconic (Germantown, NY, USA). Care for animals and all experiments followed the guidelines of the Canadian Council on Animal Care (CCAC), under protocol AUP 20-0016H, approved by animal care committees at Mount Sinai Hospital (MSH) or Toronto Centre for Phenogenomics. Oocyte-specific deletion of *Pdss2* was achieved by crossing mice with floxed *Pdss2* allele [20] with the C57BL/6-Tg(Zp3-cre)93Know/J strain, obtained from Jackson laboratories (strain # 003651) (Bar Harbor, ME, USA).

Retired breeders (females of proven fertility) were used as a model for aging and were separated from males at the age of ~8 months. Virgin females (7–8 weeks old) were used as young controls. Mice were kept on 12 h ON/OFF light-dark cycle and had free access to water and food. Nine month old mice were injected with S.C. CoQ10 (0.084 mg/kg per week; Sigma Aldrich, St. Louis, MO, USA) or placebo (sesame oil), for 12 weeks. We have previously established that this dose of CoQ10 is efficiently up taken by ovaries and raises the ovarian levels by ~3 fold [6].

### 2.2. Ovulation Induction and Cumulus Cells Collection

Mice were superovulated with 5 international units IU of pregnant mare serum gonadotropin ((PMSG); NHPP, Torrance, CA, USA or ProSpec, Rehovot, Israel), and 48 h later, with 5 IU of human chorionic gonadotropin (hCG) (Sigma-Aldrich, St. Louis, MO, USA), by intraperitoneal injection. The dose of both hormones was doubled to 10 IU, for aged dams. Mice were sacrificed ~16 h, after the last injection, oviducts were removed and COCs were retrieved in a modified human tubal fluid medium, supplemented with 0.1% bovine serum albumin BSA (Irvine Scientific, Irvine, CA, USA; Sigma-Aldrich, St. Louis, MO, USA) and denuded of cumulus cells, using hyaluronidase (Sigma-Aldrich, St. Louis, MO, USA). Ovulation data from this cohort of mice were previously reported elsewhere [6]. Isolation of COCs for the glucose uptake experiments was done in the immature oocytes isolated from the ovarian follicles, 42 h after the PMSG priming.

### 2.3. Counting Cumulus Cells Per Oocyte

Cumulus cells were stripped from the ovulated oocytes, collected from the oviducts ~14–16 h after the hCG injection. Hyalouronidase solution (Sigma Aldrich, St. Louis, MO, USA) with cumulus cells and wash drops were collected, centrifuged, and resuspended in a defined volume of medium. A sample of the cumulus cells was stained with Trypan blue and counted, using a hemocytometer. The number of cumulus cells was divided by the number of retrieved oocytes, to obtain cumulus cells/oocyte. The cells (~5000/sample from individual females) were transferred into TRIzol and stored at −80 °C, until further use. For all the other experiments, the cumulus cells from several females (usually 3) of the same age/treatment were pooled and divided among the various experiments.

Human cumulus cells collection: The study was approved by the Mount Sinai Hospital Research Ethics Board (REB 05-0044-E). Based on the customary criteria (e.g., age, ovarian reserve, cause of infertility), ovarian stimulation with standard antagonist or short agonist protocols were optimized, individually, for each patient. Eight women under 32 years (“young”) and 4 women over 39 years of age (“old”), undergoing intracytoplasmatic sperm injection were included in this study. The medium used for oocyte stripping was collected and pooled, and the cells (~20,000) were transferred into TRIZOL solution and stored at −80 °C, for further study.

### 2.4. Quantitative RT-PCR

Total RNA was isolated from TRIzol (Thermo Fisher, Mississauga, ON, Canada), following the manufacturers protocol for small number of cells, using glycogen as a carrier. To remove any residual DNA, pellets were dissolved in water and digested with amplification grade DNAaseI (Sigma Aldridge, St. Louis, MO, USA). cDNA was synthesized using RevertAid First strand Synthesis Kit (Thermo Fisher, Mississauga, ON, Canada), using oligo dT primers. Expression levels of transcripts were assessed by qPCR assay performed in the Mastercycler^®^ (Eppendorf, Mississauga, ON, Canada), using SYBR Green PCR mix (Applied Biosystems, Foster City, CA, USA or Wisent, Saint-Jean-Baptiste, QC, Canada). Amplification conditions for each primer set was optimized for efficiency. Dissociation curves at the end of the reaction were checked for each sample. Fold changes using ΔΔCΤ were generated using β-actin as a housekeeping gene. Primer sequences are listed in Supplementary Table S1.

### 2.5. Immunostaining

Stripped pooled cumulus cells were either immediately fixed in a 10% buffered formalin and used for Caspase 3-staining and terminal deoxynucleotidyl transferase dUTP nick end labeling (TUNEL), or cultured overnight in a Dulbecco’s Modified Eagle Medium (DMEM) medium with 10% FCS, on glass coverslips, followed by fixation in the PHEM fixative (80mM PIPES, 5mM EGTA, 1mM MgCl_2_, 25mM HEPES at pH of 7.2, 3.7% formaldehyde, 0.1% Triton X-100) for assessment of the COQ6 and PDSS2 immunostaining. Primary antibody-included, non-cleaved caspase 3 (1:200; #9661, Cell Signaling, Danvers, MA, USA), PDSS2 (1:200, 13544-1-AP), and COQ6 (1:100, 12481-1-A, both from Proteintech; Rosemont, IL, USA), followed by incubation with donkey anti rabbit Alexa 594 or Alexa 488, respectively (Molecular Probes, Thermo Fisher, Waltham, MA, USA). DNA was counter-stained with 4′,6-diamidino-2-phenylindole (DAPI). Imaging was performed using the WaveFX Spinning Disk Confocal Laser-Scanning Microscope (Leica DMI6000B; Quorum, Guelph, ON, Canada). Fluorescent intensity was assessed per cell and all groups were imaged with constant exposure times, for comparison of signal intensity using Volocity version 5.2.2 (Improvision Limited, Coventry, UK).

### 2.6. TUNEL Staining

DNA fragmentation of the cumulus cells was measured, as previously described [21], except for a source change of the TdT enzyme (Roche, Mississauga, ON, Canada), dUTP (Fermentas, Thermo Fisher. MA, USA), and Enzyme Buffer #4 (NEB, Whitby ON, Canada). For the TUNEL and Caspase 3 staining, the number of positive cells over the total number of cells were counted and expressed as a percentage of the total pool of the analyzed cells.

### 2.7. MitoTracker Red and Glucose Uptake Staining

Respiring mitochondria were examined by culturing cumulus cells in an Minimal Essential Medium (MEM), supplemented with BSA, overnight, on glass slides and staining with MitoTracker Red CMXROS (Molecular Probes, Thermo Fisher, Waltham, MA, USA), at the concentration of 125 nM, for 30 min. For validation of signal specificity, the cells were treated with an inhibitor of complex III (4 μM antimycin, Sigma Aldrich, St. Loius. MO, Canada), and the results of the two independent experiments are shown in Supplementary Figure S1. Cells were imaged similarly, as described for the IHC experiments. For glucose uptake staining, freshly isolated COCs were cultured with 100 μM 6-(N-(7-Nitrobenz-2-oxa-1,3-diazol-4-yl)amino)-6-deoxyglucose (6-NBDG, Setareh Biotech, Eugene, OR, USA), in an Human Tubal Fluid (HTF) medium (Life Global, Guelph, ON, Canada) for 20 min at 37 °C. After three washes, the COCs were transferred to 20 uL drops of modified Human Tubal Fluid (mHTF) medium (Life Global, Guelph, ON, Canada) on bottom glass plates (MatTek, Ashland, MA, USA), and imaged using the WaveFX Spinning Disk Confocal Laser-Scanning Microscope (Leica DMI6000B; Quorum, Guelph, ON, Canada). Fluorescent intensity was taken from 3–4 sites, within the cells of corona radiate, based on a protocol described [18], and all groups were imaged with constant exposure times. The Volocity version 5.2.2 (Improvision Ltd., Coventry, UK) software was used to determine the mean fluorescent intensity.

### 2.8. Progesterone

Cumulus cells obtained from stripped ovulated oocytes were collected, counted and plated on 96 well plates, at density of 5000 cells/well in an MEM medium supplemented with 10% FCS. Medium was collected 48 h later, and used for progesterone analysis, using the Immulite Automated Immunoassay Analyzer (DPC Immulite, Diagnostic Products Corporation, Fremont, CA, USA) Progesterone assay, with a Calibration Range: 0.2–40 nmol/L.

### 2.9. Statistical Analysis

All results are given as mean ± SEM and statistical significance was determined using the SigmaPlot 11 (Systat Software Inc., San Jose, CA, USA). All data were tested for normality/variance and, based on the outcome, parametric or non-parametric tests were chosen. Comparison of gene expression, immunostaining of Pdss2 in two aging groups and cumulus cell number in Pdss2-deficient model, and antimycin treatment in cells, was analyzed, using Mann U Whitney Rank Sum and *t*-tests for the ovulation rates. *t*-test with Welch’s correction was used for immunostaining of the CoQ6. The tests used for comparison of three groups were as follows—one-way ANOVA followed by Holm-Sidak post-hoc test (Cumulus cell number per oocyte; Progesterone), chi-square analysis for proportions (percentage of cell death and caspase 3), or Kruskal-Wallis One-Way Analysis of Variance on Ranks followed by Dunn’s Comparison (Mitotracker Red; and Glucose). Results were considered to be statistically significant, if *p* < 0.05.

## 3. Results

**Figure 1:**
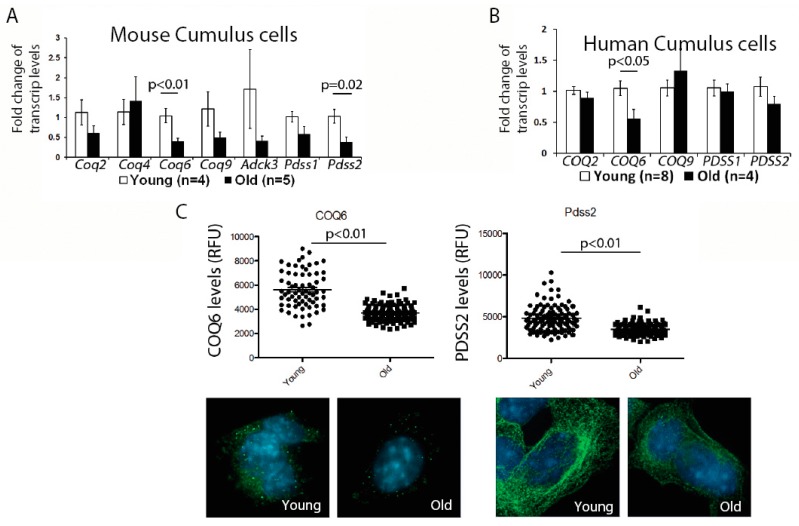
Expression of genes involved in the Co-enzyme Q (CoQ) synthesis, in young and old cumulus cells. (**A**) Fold change in the mRNA levels in the cumulus cells, with aging. Most CoQ10 synthesis gene expression, decreased with aging, with significant impact on the *Coq6* and *Pdss2*. Cumulus cells were stripped from the ovulated oocytes and pooled, per female, from young (*n* = 4) and old mice (*n* = 5). β-actin was used as a housekeeping gene. (**B**) Fold change in mRNA levels in the CoQ10 synthesis genes, from human cumulus cells pooled from several oocytes of the same patient. The cells were striped from the oocytes, prior to Intra Cytoplasmic Sperm Injection ICSI, pooled, and analyzed, similar to murine cumulus cells. Significant decrease for the CoQ6 was observed. (**C**) Immunocytochemistry with anti-CoQ6 and Pdss2 antibodies in cumulus cells from young and old mice. Reduced levels of both of these proteins in the cumulus cells, with aging, was observed. Values represent the mean fluorescence units ± SEM measured, per cell, where each dot represents a fluorescent intensity per cumulus cell, obtained by pooling cells from three different females, analyzing 60–100 cells per group (magnification 100×).

First, we examined the expression of genes involved in the CoQ10 synthesis pathway in the murine cumulus cells. While there was some variability in the expression of all transcripts examined, possibly reflecting biological variability of aging, we observed a significant reduction for the two transcripts—*Coq6* and *Pdss2*; (Figure 1A). We, next, confirmed that the protein levels for both of these enzymes were also significantly decreased (Figure 1C). Similar to mouse, human cumulus cells obtained from older patients also had a significantly lower expression of the *COQ6* transcript, while changes in the *PDSS2*, did not reach significance (Figure 1B). These results suggest that a suboptimal CoQ environment might exist in both the aged oocytes and the cumulus cells.

**Figure 2:**
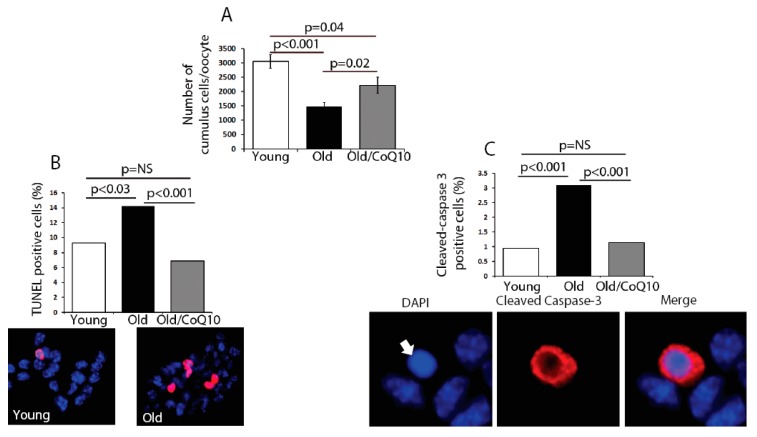
Aging impacts cumulus cell number via regulation of apoptosis. (**A**) Number of cumulus cells per oocyte declined with aging, but this was significantly improved after CoQ10 supplementation. Pooled cumulus cells from the same mouse were counted and divided by the number of ovulated oocytes, per female, to obtain the number of cells per oocyte. Values represent mean ± SEM from young (*n* = 15), old (*n* = 10), and old-CoQ10 mice (*n* = 8), per oocyte, per female. (**B**) Proportion of TUNEL and cleaved caspase-3-positive cumulus cells. Approximately 1,000 cells were counted in each category and these were generated by pooling cumulus cells from oocytes from three different females. Increased apoptosis of cumulus cells, with aging, with a protective effect of CoQ10. Representative images are shown below the graph (DAPI—blue, TUNEL—red). (**C**) Proportion of cleaved caspase-3 positive cells in each age category: images below demonstrate apoptotic cumulus cell (arrow), with the picnotic nucleus-stained positive for cleaved caspase-3 obtained from old females, as an example of staining (magnification 400×). NS: not significant.

In order to determine if age could compromise the cumulus cell physiological function, we explored several biological outcomes. The number of cumulus cells surrounding each oocyte was reduced in old mice (Figure 2). This decreased number of cumulus cells coincided with an increased rate of apoptosis, determined by an increased number of TUNEL-positive and Caspase-3-positive cells (Figure 2). CoQ10 supplementation decreased the rates of cell death and significantly increased the number of cumulus cells per oocyte.

**Figure 3:**
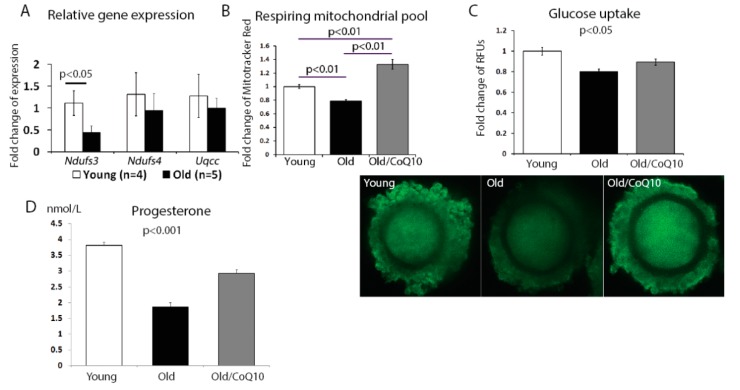
Reduced cumulus cells metabolism function with aging. (**A**) Fold change of the mRNA levels of genes involved in oxidative phosphorylation, with a significant reduction of *Ndufs3* expression in cumulus cells from old mice. Data shown were generated from the same samples as those in Figure 1 A. (**B**) Mitotracker Red signal measured in live cumulus cells from a merged pool of cells of numerous oocytes, of at least three females. Aging significantly decreased the mitochondrial respiring pool and CoQ10 supplementation increased the respiring mitochondria. The values are expressed as mean ± SEM of the fold change in fluorescence units per cell, compared to the young group (*n* = 192 for young; *n* = 208 old; and *n* = 33 for CoQ10, magnification 400×). (**C**) Glucose uptake by cumulus cells is reduced with aging, but can be improved by CoQ10 supplementation. Images below demonstrate the cumulus–oocyte complexes at the germinal vesicle stage, stained with florescent glucose (6-NBDP), and are expressed as fold change of random fluorescence units, adjusted to the young group (*n* = 65 regions from 20 young cumulus–oocyte complex (COCs), *n* = 49 regions from 15 COCs for old, and *n* = 44 regions from 14 COCs from Old/CoQ10; magnification 200×). (**D**) Progesterone production is decreased in aged cumulus cells and is restored by in vivo treatment of females with CoQ10. Cumulus cells were pooled from at least three females, for each treatment group, and plated on 96 well plates with *n* = 18 young, *n* = 9 for old, and *n* = 8 for old/CoQ10.

The aging process in cumulus cells also reduced the mitochondrial activity. We found a significant decrease in the expression of the nicotinamide adenine dinucleotide (NADH)-coenzyme Q reductase subunit 3; *Ndufs3* (Figure 3A); however, no change was observed for subunit 4 (*Ndufs4*) or ubiquinol-cytochrome c reductase complex chaperone (*Uqcc1*). In addition, the respiring mitochondrial pool declined in aged cumulus cells, but increased in response to the CoQ10 treatment (Figure 3B).

Since one of the key functions of the cumulus cell is to fuel the oocyte metabolism, we investigated if glucose uptake was affected by aging. Glucose uptake decreased in cumulus cells from old, in comparison to young mice, and this decrease was improved by the CoQ10 supplementation (Figure 3C).

Finally, as the mitochondria are responsible for steroid hormone production, we analyzed progesterone production by cumulus cells maintained in a culture for 48 h. Consistent with previous reports, progesterone production was decreased in the aged group and was significantly improved by the CoQ10 in vivo administration (Figure 3D).

We have previously showed that *Pdss2* is required for a proper mitochondrial function in the oocytes, as oocyte-specific deletion of this CoQ gene created mitochondrial metabolic dysfunction and recapitulated many oocyte phenotypes linked to aging [6]. In order to establish if the aging cumulus cell number could be a consequence of *Pdss2* deficiency in the oocyte, we counted the number of cumulus cells in the ovulated oocyte complexes of the wildtype and *Pdss2*-depleted oocytes. While *Pdss2* deficiency significantly decreased the number of ovulated oocytes, no impact was detected on the number of cumulus cells per oocyte (Supplementary Figure S2).

## 4. Discussion

In this study, we demonstrated that the aging process includes a reduced CoQ synthesis genes expression in cumulus cells from aged mice and humans. Moreover, in aged mice we found a reduced number of cumulus cells per oocyte, secondary to increased apoptosis. We also observed a reduced mitochondrial respiring pool and diminished glucose uptake by cumulus cells. CoQ10 supplementation mitigated these aging-associated phenotypes, suggesting that cumulus cells, similar to oocytes, can benefit from the CoQ10 supplementation therapy.

The aging process in the female reproductive organs involves an increased rate of apoptosis [22]. A previous study suggested that the competence of the human oocyte to undergo fertilization was associated with the cumulus cell DNA fragmentation, a hallmark of cell death [23]. We confirmed an increased apoptosis of cumulus cells, with aging, as well as its prevention with CoQ10 supplementation. We proposed that CoQ10 supplementation, by increasing the number of cumulus cells surrounding each oocyte, led to an increased developmental competence and improved the reproductive performance of aged females. Moreover, the observation that CoQ6 and Pdss2 expression was reduced in the cumulus cells of aged females, suggests that the local CoQ decline might be involved in the aging process, at least in some somatic compartments of the aged ovary. The total ovarian extracts contained relatively large amount of CoQ9, the most abundant form of rodent CoQ, as well as some CoQ10 [24]. However, we do not know what cell types contribute most to this pool and if aging alters their composition. It is clear that ovaries, as an organ, can efficiently uptake dietary CoQ10 [6,24], but it is less clear what cell types are the primary targets of its action.

We also considered the possibility that cumulus cell number in aged oocytes was decreased due to the *Pdss2* decrease in the aged oocytes. However, *Pdss2* deficient oocytes, which recapitulated many hallmarks of biological aging, were not surrounded by fewer cumulus cells, pointing to a cell autonomous defect in this somatic compartment.

Metabolic changes occurred with aging in human, as evidenced by the altered cumulus cell molecular signature, with an enrichment of pathways involved in oxidative phosphorylation ((OP); NDUFA1, UQCRC1, MT-ATP6, ATP5I, and MT-ATP8), [7]. Consistent with these findings, we also observed a decrease of *Ndufs3* expression and a decline in the mitochondrial respiratory pool in cumulus cells of old mice which were restored by the CoQ10 treatment. This was also consistent with a decrease in the cumulus cell mitochondrial complex I function in older women and its improvement, after an addition of CoQ, in vitro [8]. Interestingly, the response of mitochondrial membrane potential to uncoupling agent in cumulus cells, correlated with the ovarian response to superovulation [25], indicating the relationship between the germline recruitment and mitochondrial function in the supporting somatic lineage.

Glucose metabolism in the cumulus cells is crucial in determining oocyte developmental competence [26]. A steady state level of glucose metabolism is needed to maintain both energy and the intracellular redox potential to prevent oocyte postovulatory aging [27]. Moreover, a decreased glucose uptake in the cumulus cells is associated with impaired oocyte quality in female diabetic mice [18]. In addition, modulation of glycolytic activity in bovine cumulus oocyte complexes, triggers changes in the mitochondrial activity in oocytes [28]. We used a similar approach of live imaging of glucose transport, by cumulus oocyte complexes, in aged dams and found a decrease with aging but improvement after the CoQ10 supplementation. Interestingly, cumulus cell phillopodia, which facilitate the exchange of some metabolites, are decreased in aged oocytes [29], likely impeding the communication, as well as possibly limiting the metabolic flux of nutrients. Whether the CoQ10 could improve this outcome remains to be established.

## 5. Conclusions

In summary, in an aged murine model, we identified the changes in the cumulus cells viability and physiological function. This aging process could be partially reversed by the CoQ10 supplementation. These encouraging results further strengthened the need for controlled clinical studies of the effect of CoQ10 supplementation in humans.

## Supplementary Materials

The following are available online at https://www.mdpi.com/2076-3921/8/3/58/s1, Figure S1. Specificity of Mitotracker signal in granulosa cells, Figure S2. Cumulus cell number and ovulation rate in females with Pdss2 deficient oocytes, Table S1: Primer list with qRT-PCR conditions.
