# Supplementary material for: Co-Enzyme Q10 Supplementation Rescues Cumulus Cells Dysfunction in a Maternal Aging Model
Source: Antioxidants (Basel). 2019 Mar 8;8(3):58. doi: 10.3390/antiox8030058 (PMC6466589; doi:10.3390/antiox8030058)
Supplement: Supplementary file 1 [file antioxidants-08-00058-s001.pdf]

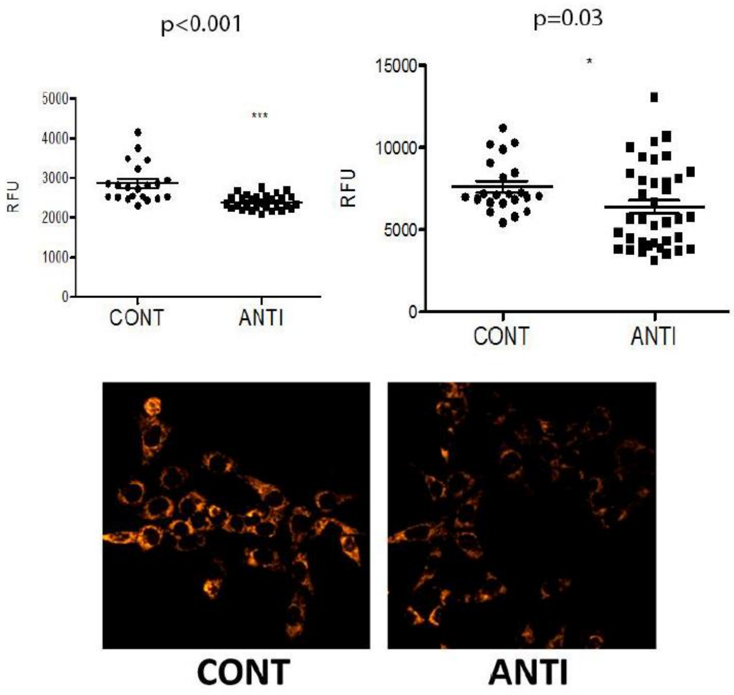

Figure S1. Specificity of Mitotracker signal in granulosa cells.

Cells were co-treated with inhibitor of complex III, antimycin for 30 min validating signal specificity. Each point represents a cell. Significantly decreased fluorescent intensity was observed in two independent experiments.

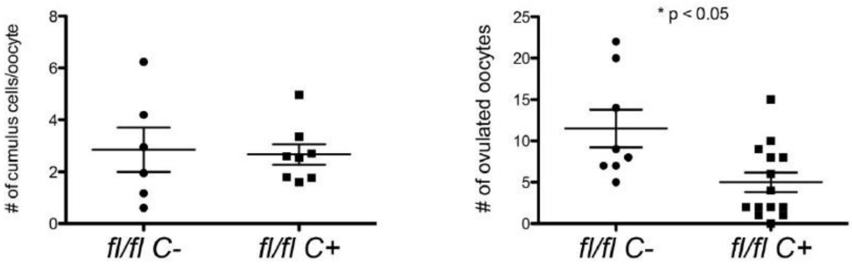

Figure S2. Cumulus cell number and ovulation rate in females with Pdss2 deficient oocytes.

Pdss2 deficiency in oocytes due to deletion by ZP3Cre recombinase in primary follicles does not change number of cumulus cells per oocyte but leads to significant decrease of ovulation rate. Data shown are number of cells per oocyte per female (left panel) or number of total ovulated oocytes per females (right panel); genotype Pdss2 ff C (WT) and ff C+ (KO).

Table 1. Primer list with qRT-PCR conditions.

Name of gene	Primer Sequence	Ref Sequence	Amplicon size	% efficiency	annealing temperature
Bact	cggttccgatgccctgaggctctt<br>cgtcacacttcatgatggaattga	NM_007393.5	99	100	62.5
Coq2	atccacagagccgaggact<br>tcagctcgtctgctcacttc	NM_027978.2	158	94	61.5
Coq4	ctgacttccgtccccaaga<br>acatctccccaaaccatcac	NM_178693.4	148	100	62.5
Coq6	ggctgctactgacctgttga<br>aggagacactgcgttcgtg	NM_172582.3	99	94	60
Coq9	ctgggaggagcagcagtaga<br>ttgtgatttccagccttgtg	NM_026452.2	123	104	62.5
Pdss1	ttttaccagactgtgcctaatg<br>ccgaaaaactttgccaataaa	NM_019501.3	126	101	61.5
Pdss2	caggagagcttttggtgatg<br>tccttctcgttttgtttgtagtg	NM_027772.2	133	99	60
Ndufs3	ttatggcttcgagggacatc<br>attcttgtgccagctccact	NM_026688.2	118	94	62.5
Ndufs4	tgccgagtatgacgtgtctc<br>cttgcataggtccagcgaat	NM_026610.1	155	100	62.5
Uqcc1	tactcctgtccccaggtcac<br>cgtaaagagaggccaagtgc	NM_018888.4	201	104	61.5
BACT	gcgttgttacaggaagtcccttg<br>ctatcacctcccctgtgtgga	NM_001101.4	100	99.7	60
COQ2	ttttcttgggggacagctaa<br>ggctagttgaggccagtatga	NM_015697.8	149	89	60
COQ6	ctaagcagttggaggctgtgt<br>atttggtctggaaggtgctg	NM_182476.2	150	100	58
COQ9	cgaggaggaggaggactatga<br>ccttctgcaatcgcctct	NM_020312.4	110	95	60
PDSS1	atcatgcgacggttcagttt<br>ggcgaggtaggttgtttgtt	NM_014317.4	93	105	58
PDSS2	ttggtggtgctccttatctct<br>catgagtagatcccactgacc	NM_020381.3	92	104	60
